# Direct solution-phase synthesis of 1T’ WSe_2_ nanosheets

**DOI:** 10.1038/s41467-019-08594-3

**Published:** 2019-02-12

**Authors:** Maria S. Sokolikova, Peter C. Sherrell, Pawel Palczynski, Victoria L. Bemmer, Cecilia Mattevi

**Affiliations:** Department of Materials, Imperial College London, London, SW7 2AZ UK

## Abstract

Crystal phase control in layered transition metal dichalcogenides is central for exploiting their different electronic properties. Access to metastable crystal phases is limited as their direct synthesis is challenging, restricting the spectrum of reachable materials. Here, we demonstrate the solution phase synthesis of the metastable distorted octahedrally coordinated structure (1T’ phase) of WSe_2_ nanosheets. We design a kinetically-controlled regime of colloidal synthesis to enable the formation of the metastable phase. 1T’ WSe_2_ branched few-layered nanosheets are produced in high yield and in a reproducible and controlled manner. The 1T’ phase is fully convertible into the semiconducting 2H phase upon thermal annealing at 400 °C. The 1T’ WSe_2_ nanosheets demonstrate a metallic nature exhibited by an enhanced electrocatalytic activity for hydrogen evolution reaction as compared to the 2H WSe_2_ nanosheets and comparable to other 1T’ phases. This synthesis design can potentially be extended to different materials providing direct access of metastable phases.

## Abstract (web summary)

1T’ phases of transition metal dichalcogenides show promise for electrocatalysis, energy storage, and spintronic applications but are difficult to obtain. Here the authors synthesize 1T’ WSe2 few-layered nanosheets by kinetically-controlled colloidal synthesis, and test their electrocatalytic activity.

## Introduction

Atomically thin transition metal dichalcogenides (TMDs) have been attracting enormous attention in the past decades as promising materials for a variety of disparate applications ranging from flexible electronics^1^ to electrocatalysis and photoelectrocatalysis^2,3^, electrochemical actuators^4^ and energy storage devices^5^ to the recently proposed topological electronic devices^6^. The versatility of properties of TMDs is enabled by the polymorphism of their monolayers, where metal coordination changes from trigonal prismatic (2H phase) to octahedral (1T phase) and distorted octahedral (1T’ phase).

The 1T and 1T’ phases of group VI TMDs started to emerge only recently; however, compared to the semiconducting 2H phase, they have exhibited an exceptional performance for electrocatalytic hydrogen evolution and energy storage applications owing to the dramatically reduced charge transfer resistance^7^ due to their metallic nature^8^. Moreover, 1T’ WSe_2_ single layer is predicted to be a large gap quantum spin Hall (QSH) insulator suitable for application in spintronic devices^9^ both operable at ambient temperature in contrast to the 1T’ WTe_2_^10^ and benefiting from chemical stability of the material compared to that of other currently known large gap QSH insulators such as stanene^11^ and two-dimensional In-Sb compounds^12^ existing in an inert atmosphere only.

Owing to the metastable nature of the 1T and 1T’phases of group VI sulphides and selenides (MoS_2_, MoSe_2_, WS_2_ and WSe_2_), direct synthesis of these materials generally leads to formation of the thermodynamically stable 2H phase^13,14^ with a few recent exceptions of wet-chemical synthesis of the WS_2_ nanodisks^15^ and WS_2_ nanoribbons^16^ with the distorted 1T structure, however, stabilised by the charged precursor residues intercalated between the layers. This metastable 1T’ phase converts into the thermodynamically stable 2H phase once the charges are removed. The direct synthesis of the metastable 1T’ phase of high crystal quality and not stabilised by foreign species^17^ is challenging and has only been recently reported for bulk MoS_2_ and MoSe_2_ crystals^18^.

The 1T/1T’ phases are more commonly obtained via distortion of the 2H phase as a result of either electrical gating^19^ or electron transfer during chemical treatment (i.e. lithiation during exfoliation or organolithium treatment)^20^ or doping beyond degenerate level^21^ or applied mechanical strain^22^. While for MoS_2_ and WS_2_ the 1T’ phase has been reported via these treatments, there is no current evidence that electron transfer can convert the 2H phase of WSe_2_ into the 1T’. Indeed, intercalation studies with organolithium treatment followed by exfoliation show evidence of persistency of 2H WSe_2_^23^. Atomically thin WSe_2_ nanosheets prepared via wet chemical approaches or vapour deposition techniques reported so far acquired the thermodynamically stable 2H phase^13,24,25^ with the only exception of molecular beam epitaxy deposition, which led to the formation of nanometre-sized 1T’ WSe_2_ single layers^9^. Overall, there are no reported established methods to achieve 1T’ WSe_2_ over large areas or measurable quantities, although energy difference between 1H and 1T’ phases of WSe_2_ is only 0.27 eV per formula unit that suggests that the metastable 1T’ can be obtained under certain reaction conditions^26^.

Here we demonstrate a direct solution-phase synthesis of the metastable distorted octahedral structure (1T’ phase) of WSe_2_ not stabilised by charged species. We design a kinetically controlled regime of colloidal synthesis to enable nucleation and growth of 1T’ WSe_2_ branched few-layered nanosheets with high yield and in a reproducible manner. We report a full study of the atomic structure (high-resolution transmission electron microscopy (HR TEM)), the Raman characteristic peaks, chemical bonding (X-ray photoelectron spectroscopy (XPS)) and layer stacking order (powder X-ray diffraction (XRD)) proving the 1T’ nature of WSe_2_. We also found that the 1T’ phase is fully convertible into the semiconducting 2H phase upon thermal annealing at 400 °C. The 1T’ WSe_2_ nanosheets demonstrate an enhanced electrocatalytic performance for hydrogen reduction as compared to the 2H WSe_2_ as expected for the metallic phases.

## Results

### Characterisation of the colloidal 1T’ WSe_2_ nanosheets

**Fig. 1:**
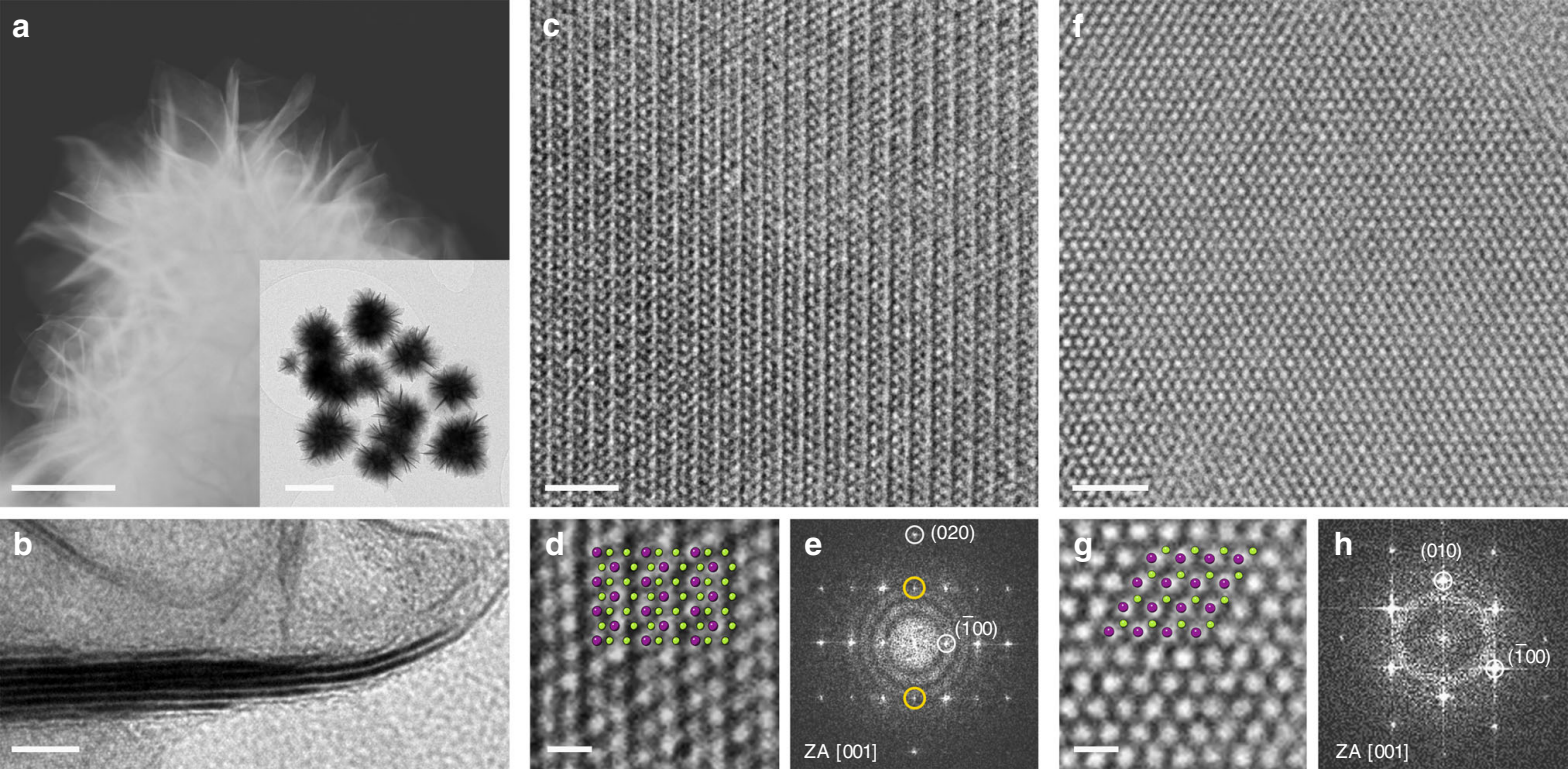
Transmission electron microscopy (TEM) characterisation of the colloidal WSe_2_ nanostructures. **a** Annular dark-field scanning TEM image of a WSe_2_ branched nanoflower illustrating ultra-thin nature of individual nanosheets (scale bar: 100 nm), inset—an overview image of an ensemble of the WSe_2_ nanoflowers (scale bar: 200 nm). **b** Side view TEM image of an individual petal thinning down to monolayer at the rim (scale bar: 5 nm). **c** High-resolution TEM image of the as-produced WSe_2_ nanosheets demonstrating a continuous 1T’ phase over a large area (scale bar: 2 nm); **d** zoomed in image (scale bar: 0.5 nm) with an overlaid crystal model of the 1T’ phase of WSe_2_ illustrating the zigzag chains of tungsten atoms; **e** fast Fourier transform (FFT) pattern of the area shown in **c**, yellow circles highlight kinematically forbidden (010) reflections. **f** High-resolution TEM image of the annealed WSe_2_ nanosheets (scale bar: 2 nm); **g **zoomed in image (scale bar: 0.5 nm) with an overlaid crystal model of the 2H WSe_2_ phase displays the uniformly spaced hexagonal lattice; **h **FFT pattern of the area shown in **f**. In the overlaid crystal models, purple circles represent tungsten atoms, and green circles represent selenium atoms

1T’ WSe_2_ nanosheets were synthesised through a solution-phase reaction between tungsten hexacarbonyl and trioctylphosphine selenide (TOP:Se) complex in hot oleic acid (OlAc) as described in the Methods section. TEM images shown in Fig. 1a reveal that under described growth conditions WSe_2_ forms well-defined flower-like nanostructures with an average diameter of 200 nm. The nanoflowers are made of multiple petals emerging from a central core region. In annular dark-field scanning transmission electron microscopy (ADF STEM) images, petal edges appear curled and ultra-thin in nature, while side view TEM images clearly demonstrate that the petals are thinning down to single layer towards the rims (Fig. 1b).

HR TEM analysis demonstrates that as-produced WSe_2_ nanoflowers are acquired in the 1T’ phase with individual petals being single crystals. Representative HR TEM images shown in Fig. 1c and in Supplementary Fig. 2 exhibit evident contrasting zigzag chains of tungsten atoms characteristic to the 1T’ structure^27^. Corresponding fast Fourier transform (FFT; Fig. 1e) can be indexed in the [001] zone axis of the monoclinic 1T’ phase of WSe_2_ (Supplementary Fig. 1). Observed in the experimental FFT patterns, (010) reflections are kinematically forbidden in the 1T’ phase and can be indicative of the antisite defects present in the few-layered nanosheets. Structural parameters *a* = 5.76 Å and *b* = 3.30 Å of the 1T’ phase of WSe_2_ were identified from the results of HR TEM imaging. It should be noted that the experimentally found lattice constant *a* is slightly smaller than the calculated equilibrium value (5.94 Å^26^) presumably due to the scrolling of flexible ultra-thin petals along the [010] direction perpendicular to the petal rim (Supplementary Fig. 3).

In contrast to a chemical vapour deposition (CVD) growth that occurs in a diffusion-mediated regime^14^ and so leads to formation of the thermodynamically stable phase only, liquid-phase growth appears to be dominated by the reaction kinetics thus allowing achieving the metastable phase. In the case of bulk WSe_2_, energy difference between 2H and 1T’ phases is as small as 0.27 eV per MX_2_^26^, suggesting that the metastable phase can be directly grown under favourable synthetic conditions such as high monomer concentration (high supersaturation), when the monomer adsorption rate exceeds the dissolution rate and a kinetically driven growth of the metastable phase nuclei is favoured^28^. At high monomer concentration, nuclei critical size is notoriously small, and we cannot exclude that stable clusters of both 1T’ and 2H ‘phases’ may nucleate in solution. Second, zigzag chains running along [010] direction of the 1T’ phase are likely to propagate faster^29^, so out of two possibly competing reactions growth of the 1T’ phase along the [010] direction might be favourable over 2H as it may minimize the total surface energy.

Synthesis temperature reported here was kept not >300 °C as higher temperatures would lead to solvent boiling and lower temperatures would inhibit any nucleation and growth to occur. The fact that high supersaturation at synthesis temperature plays a key role in determining the formation of 1T’ phase is supported by the evidence that the phase obtained is unaffected by the use of different precursors of tungsten and selenium, by changing coordinating solvent or the synthesis time (between 3 and 15 h) as summarised in Supplementary Figs. 4–6. Specifically, changing from strongly binding OlAc to a weaker coordinating oleylamine (OlAm) solvent, variation of selenium (from less active TOP:Se to more active elemental Se) and tungsten (from less active W(CO)_6_ to more active WCl_6_) precursor reactivity and additional activation of tungsten precursor by complexing with additives (hexamethyldisilazane and tetradecylphosphonic acid) consistently led to the formation of the metastable 1T’ phase and to negligible changes in the morphology. The least active tungsten carbonyl and the most active elemental selenium yield the highest-quality WSe_2_ nanoflowers. Furthermore, crystallisation in the metastable 1T’ phase with metal chains running through the structure can potentially explain the formation of branched structures themselves. Branched morphology has been reported for the 1T’ ReS_2_ nanosheets where distortion in the metal sublattice was suggested to facilitate adsorption of Re atom at the Re-Re metal bond leaving highly reactive under-coordinated chalcogen atom that served as a nucleation site for the growth in the direction perpendicular to the surface of 1T’ ReS_2_ nanosheet^30^.

### Conversion into the thermodynamically stable 2H phase

The produced WSe_2_ nanoflowers form stable suspensions in ethanol (Supplementary Fig. 7) and the 1T’ phase is found to be stable under ambient conditions (Supplementary Fig. 8) as well as under electrocatalytic operation conditions as explained within the ‘Electrochemical characterisation’ section. However, upon thermal annealing the 1T’ phase can be converted into the thermodynamically stable phase, which is the semiconducting 2H phase. Differential scanning calorimetry (DSC; Supplementary Fig. 9) shows that the 1T’ phase undergoes an irreversible phase transition at 395 °C at atmospheric pressure. TEM analysis confirms that overall branched morphology of WSe_2_ nanostructures is preserved without any visible diminishing in lateral size or increase in thickness (Supplementary Fig. 10). HR TEM imaging proves that the material adopts a hexagonal structure with uniform interplanar spacing of 2.84 Å corresponding to the (100) planes of the semiconducting 2H WSe_2_ (Fig. 1f–h).

**Fig. 2:**
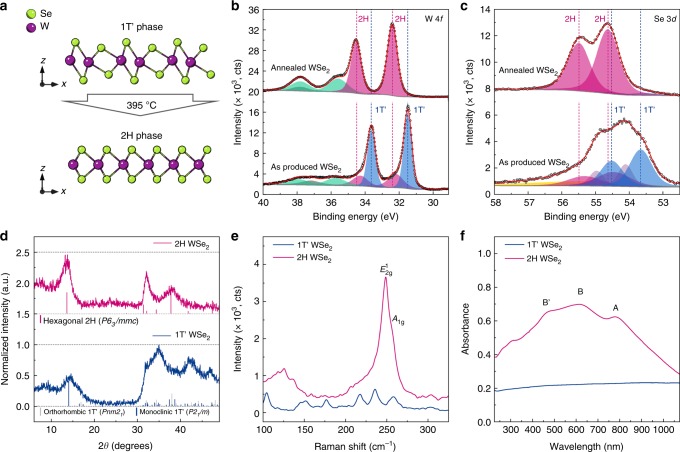
1T’ to 2H phase transformation. **a** Schematic representation of the 1T’ to 2H structural phase transition occurring upon thermal annealing. **b**, **c** X-ray photoemission spectra (XPS) of the W 4*f* and the Se 3*d* core-level electrons before (bottom row) and after (upper row) phase conversion, respectively. In XPS spectra, doublets corresponding to the 1T’ phase of WSe_2_ are given in blue, 2H phase of WSe_2_ in pink, WO_3_ in green, 5*p*_3/2_ component of WSe_2_ in grey, yellow and violet doublets in the Se 3*d* spectrum are assigned to the coordinated surface selenium. **d** X-ray diffraction patterns, **e** Raman spectra, and **f** Ultraviolet–visible absorption spectra of the as-produced and the annealed (400 °C) WSe_2_ nanoflowers. Raman spectra are presented in the same scale but are offset for clarity

The evidence of the 1T’ to 2H phase conversion was additionally provided via powder XRD, XPS, Raman and optical (ultraviolet–visible (UV-vis)) absorption spectroscopy. Prior to the XPS experiments, as-produced WSe_2_ nanoflowers were dried at 200 °C in an argon atmosphere in order to remove unreacted organic species trapped in the branched WSe_2_ structures; under these conditions, the material does not undergo a phase transition (Supplementary Fig. 11). The XPS spectrum of the 4*f *W core electron shell of the as-produced WSe_2_ nanoflowers is shown in Fig. 2b. The apparent doublet of the 4*f *W^+^^4^ peaks (31.49 and 33.63 eV) was found to be significantly shifted from the reported positions of the 2H WSe_2_ and was assigned to the 1T’ phase. Similar shifts to lower binding energies have been reported for the 1T phase of MoS_2_ and MoSe_2_ that was produced upon *n*-butyllithium treatment of the CVD-grown 2H polycrystalline films^20^. The shift to the lower binding energies can be attributed to the change in the metal coordination and to a slight increase of the W-Se bond lengths from 2.531 Å in the 2H phase to 2.66 Å calculated for the 1T’ phase. Minor fraction of the 2H phase (32.22 and 34.30 eV) and traces of oxidized tungsten (35.74 and 37.92 eV) are also present. Thermal annealing at 400 °C in argon atmosphere led to a complete conversion of the 1T’ phase into the hexagonal 2H phase with the 4*f *W^+^^4^ peaks found at 32.40 and 34.58 eV, while the lower energy 1T’ 4*f *W^+^^4^ doublet was nearly absent (<2 at.%). A minor increase of the oxide content occurs during the thermal annealing as indicated by an increase in intensity of the 4*f *W^+^^6^ doublet (<25 at. %). Similarly, two types of Se^−2^ atoms are found in the as-produced WSe_2_ nanoflowers (Fig. 2c). Broad unresolved spectrum of the 3*d* Se core level electrons was fitted with four doublets with the 1T’ Se^−2^ doublet found at 53.60 and 54.46 eV and the 2H Se^−2^ doublet at 54.63 and 55.49 eV. After annealing, only the 2H doublet is observed in the 3*d* Se spectrum.

Analogously to other distorted octahedral polymorphs of group VI TMDs, bulk 1T’ phase of WSe_2_ belongs to either orthorhombic or monoclinic syngony depending on the stacking order of the octahedrally coordinated monolayers as reported for the T_d_ (1T’) WTe_2_^10,31^ and MoTe_2_^32^ (space group *Pnm2*_*1*_) or the 1T’ MoTe_2_^33^ (space group *P2*_*1*_*/m*), respectively. The experimental XRD pattern of the as-produced WSe_2_ is shown in Fig. 2d. Peaks in the diffractogram are broadened owing to the small size of WSe_2_ nanoflowers, whereas presence of strong (002) reflections suggests that the bulk of WSe_2_ nanoflowers is a few-layered material. However, the overall symmetry differs significantly from that of the annealed WSe_2_ nanoflowers acquiring the 2H phase and demonstrating (100) and (103) reflections becoming prominent; their positions are in a good agreement with the standard reference positions of the bulk WSe_2_ (JCPDS 38–1388). Reference diffraction patterns of the 1T’ phase of WSe_2_ were simulated as described in the Methods section and are presented in Fig. 2d along with the experimental diffractogram. In contrast to the orthorhombic phase, the monoclinic phase possesses inversion symmetry that implies a different set of the reflection conditions. In particular, all (00*l*) reflections are allowed in the monoclinic phase, whereas only (00*l*) where *l* = 2*n* reflections are allowed in the orthorhombic phase. We suggest that a broad reflection found at 7 degrees 2*θ* in the experimental small-angle diffraction pattern of the as-produced WSe_2_ (Supplementary Fig. 12) can be assigned to the (001) planes and thus be indicative that the 1T’ phase of WSe_2_ belongs to the monoclinic syngony. This assumption is also supported by the evidence that the orthorhombic 1T’ phase of MoTe_2_ is a low-temperature polymorph that can be only obtained via cooling the monoclinic 1T’ MoTe_2_ down to cryogenic temperatures^32^.

Raman spectrum of the 1T’ phase of WSe_2_ shows a clearly distinct set of vibration modes of extremely low intensity compared to the semiconducting 2H phase (Fig. 2e). Low intensity of Raman signal was experimentally observed in case of *n*-butyllithium-treated WSe_2_ monolayers and was attributed to poor interaction of the metallic phase with light^34^. In the Raman spectrum of the annealed WSe_2_ nanosheets, a pair of unresolved peaks at 248.6 and 258 cm^−1^ corresponding to the in-plane $E_{{\mathrm{2g}}}^1$ and out-of-plane *A*_1g_ vibration modes of the hexagonal WSe_2_ is observed. The absence of the $B_{{\mathrm{2g}}}^1$ peak at 304 cm^−1^ is indicative of the ultra-thin nature of the colloidal WSe_2_ nanosheets. To date, there is no information on the vibrational modes of the 1T’ phase of WSe_2_, although overall lower symmetry of the 1T’ phase suggests a wider set of the Raman active modes. Experimental Raman spectrum of the 1T’ phase shows 6 resolved peaks at 104.5, 149, 177, 218, 236.3 and 258 cm^−1^. Similar changes in Raman spectra were observed in 1T’ MoS_2_ with the appearance of J_1_, J_2_, and J_3_ transitions^18^.

Similar conclusions on metallic character of the 1T’ phase can be drawn from the results of optical absorption spectroscopy showing featureless metallic scattering of the as-produced WSe_2_ (Fig. 2f). Thermal annealing is accompanied by the appearance of excitonic transitions of the semiconducting WSe_2_ red-shifted from the monolayer positions that are in a good agreement with the few-layered nature of the thus synthesised material discussed above.

**Fig. 3:**
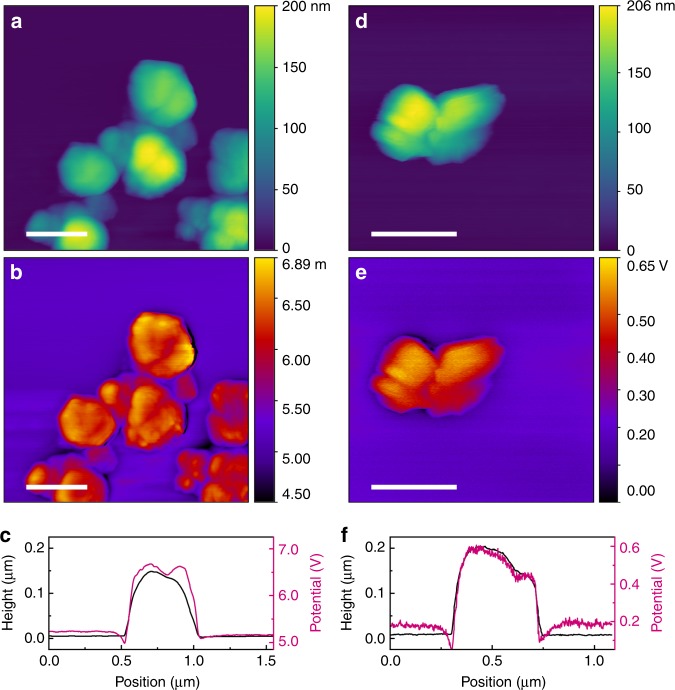
Electronic properties of the 1T’ and 2H WSe_2_ nanosheets. **a** Tapping mode atomic force microscopic (AFM) image, **b** Kelvin probe force microscopic (KPFM) image and (**c**) height profile (black) and the corresponding contact potential difference variation (purple) of the as-produced 1T’ WSe_2_ nanoflowers assembled on a p-doped Si wafer. **d** Tapping mode AFM image, **e** KPFM image and **f** the line scans of the annealed 2H WSe_2_ nanoflowers. Scale bars: 500 nm

Electronic properties of the few-layered 1T’ WSe_2_ remain majorly unexplored. In order to examine the electronic structure of the as produced 1T’ and the annealed 2H WSe_2_ nanoflowers, Kelvin probe force microscopy (KPFM) study was carried out (Fig. 3). Work functions of the 1T’ and 2H phases measured based on the known work function of p-doped Si used as a reference were found to be close to 2.6 and 4.2 eV, respectively. These values are in a good agreement with the measured work functions of the semiconducting 2H (4.08 eV) and produced through potassium surface functionalisation metallic 1T’ (2.36 eV) WSe_2_ bilayer^35^.

### Electrocatalytic activity of the 1T’ WSe_2_ nanosheets

**Fig. 4:**
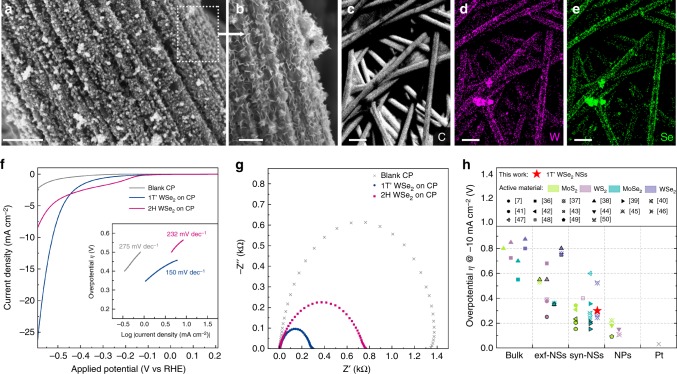
Electrocatalytic performance of the 1T’ and 2H WSe_2_ nanosheets assembled on carbon paper (CP). **a**, **b** Scanning electron microscopic images of the vertically aligned 1T’ WSe_2_ nanosheets densely covering the available surface of carbon fibres (**a**, scale bar: 1 µm), while maintaining the ultra-thin morphology of the individual petals (**b**, scale bar: 500 nm). **c**–**e** Elemental (C, W and Se) maps of the WSe_2_ nanosheets grown directly on CP illustrating the achieved uniform coverage of the functional substrate (scale bars: 20 µm). **f** Polarisation curves of the 1T’ and 2H WSe_2_ nanosheets grown on CP, inset shows corresponding Tafel slopes. **g** Nyquist plots of the 1T’ and 2H WSe_2_ electrodes. **h** Comparison with the reported group VI TMD electrocatalysts; comparative data points are taken from refs. ^7,43,39,38,44,41,45,46,42,47,37,48,49,40,50,51^ and divided into four categories based on the catalyst morphology: bulk material, mechanically and chemically exfoliated nanosheets (exf-NSs), nanosheets produced via wet-chemical approaches (syn-NSs), nanoparticles and clusters (NPs); data points with black borders correspond to the metallic 1T/1T’ phase of group VI TMDs. Comparison plot with catalyst mass loading stated for each reference data point and 1T’ and 2H WSe_2_ nanoflowers reported in this work is presented in Supplementary Fig. 16

To prove the metallic behaviour of the 1T’ phase of WSe_2_, we tested as-produced nanosheets as an electrocatalyst for hydrogen evolution reaction. In analogy with WS_2_, MoS_2_ and MoSe_2_ in their metallic phase, few-layered 1T’ WSe_2_ nanosheets are expected to exhibit an enhanced catalytic activity towards hydrogen reduction compared to their semiconducting counterparts^23^. The advantage of using a colloidal phase synthesis is the capability to grow 1T’ WSe_2_ nanosheets directly on an arbitrary conductive electrode including various carbon-based substrates and metal foils. As an approach to assemble electrocatalyst on a conductive supporting platform, direct growth can be beneficial since it allows utilising large surface area of conductive substrates such as carbon paper (CP), carbon foil (CF) and gold foil (Supplementary Fig. 13). Additionally, direct growth ensures intimate contact between the electrocatalyst and support material, leading to excellent adhesion and electrical connection to the substrate. The improved electrical connection was proved to play a crucial role in catalytic performance of the semiconducting MoS_2_ through the contact resistance engineering^36^. Scanning electron microscopy (SEM) imaging of the 1T’ WSe_2_ formed on carbon paper (Fig. 4a, b) shows vertically aligned nanosheets uniformly covering the carbon fibres. The direct growth on conductive carbon paper results in a well-adhered coating stable under electrochemical testing conditions (Supplementary Fig. 14).

Assembled on carbon paper, 1T’ WSe_2_ nanosheets were tested as an electrocatalyst for hydrogen reduction as produced, whereas 2H WSe_2_ nanosheets were obtained via thermal annealing of the electrodes at 400 °C in argon atmosphere. It should be mentioned that thermal annealing did not affect the morphology of the WSe_2_ nanosheets as demonstrated in Supplementary Fig. 14. Mass loading of active material per electrode was as low as 40 μg cm^−2^ and was determined via inductively coupled plasma emission spectroscopy (ICP ES). Electrochemical measurements were performed in a standard three-electrode cell configuration in 1 M H_2_SO_4_ electrolyte. Polarisation curves of bare CP and CP covered with the 1T’ or 2H WSe_2_ nanosheets are shown in Fig. 4f. 1T’ WSe_2_ nanosheets demonstrated a good catalytic activity with an overpotential of 510 mV at a benchmarking current density of −10 mA cm^−2^ and a Tafel slope of 150 mV dec^−1^ outperforming the semiconducting 2H counterpart (640 mV and 230 mV dec^−1^, respectively). We should remark that the mass loading of active material has a direct impact on the current density at the working electrode and, as a consequence, on the measured overpotential at the benchmarking current density commonly used as a catalyst performance metrics^37^. Therefore, considerable improvements in performance for HER are possible to demonstrate at higher catalyst loadings. In order to prove this, we deposited the 1T’ WSe_2_ nanosheets via drop casting to obtain a higher mass loading of 250 μg cm^−2^, which is closer to a typical mass loading reported in literature. We could thus demonstrate −10 mA cm^−2^ current density achieved at only 300 mV overpotential (Supplementary Fig. 15).

Electrochemical impedance spectroscopy (EIS) conducted on the as-produced (1T’) and annealed (2H) WSe_2_ electrodes confirmed better conductivity of the 1T’ phase as depicted in a significant decrease in charge-transfer resistance from 750 Ω in the semiconducting 2H WSe_2_ to 280 Ω in the metallic 1T’ WSe_2_ (Fig. 4g). Further, the decreased charge transfer resistance post-growth is indicative of the excellent electrical connection attained by direct growth on the CP substrate. The enhanced electrocatalytic performance of the 1T’ polymorph can be attributed to a faster charge-transfer kinetics in this metallic phase as compared to the semiconducting 2H phase. However, activation of catalytic sites in the basal plane of the 1T’ phase can also facilitate catalysis as was proposed in case of chemically exfoliated strained 1T WS_2_^7^.

The catalytic performance of the colloidal 1T’ WSe_2_ nanosheets reported here is comparable to that of chemically exfoliated MoS_2_ and WS_2_ (1T phases)^38^ and better than that of exfoliated 2H MoS_2_ and WSe_2_^39^ and synthesised 2H WS_2_^40^, MoSe_2_^41^ and WSe_2_^42^ nanosheets (Fig. 4h).

To conclude, our designed synthesis process is scalable and offers direct deposition of materials over functional substrates. We shed light on the structure properties relationship of the 1T’ WSe_2_, which is a challenging metastable phase to achieve, uncovering its electronic properties and catalytic activity for hydrogen evolution reaction. This synthesis design can potentially provide a general tool to address the direct synthesis of metastable phases of materials.

## Methods

### Materials and stock solutions

Tungsten carbonyl (W(CO)_6_, 99.99%, Aldrich), selenium (Se, 99.99%, Aldrich), trioctylphosphine (TOP, technical, 90%, Aldrich), OlAc (technical, 90%, Aldrich), acetone (ACS grade, VWR), and ethanol (ACS grade, VWR) were used as received without further purification. In all, 1 M trioctylphosphine selenide (TOP:Se) stock solution was prepared by dissolving 5 mmol of selenium powder in 5 ml of TOP at room temperature under a nitrogen flow.

### Synthesis of the 1T’ WSe_2_ nanosheets

In all, 0.06 mmol (20 mg) of tungsten carbonyl was placed into a three-neck flask containing 8 mL of OlAc degassed prior to the synthesis. The mixture was heated to 200 °C under vacuum and held at this temperature until complete dissolution of tungsten carbonyl precursor. After a clear pale-yellow solution was formed, the flask was flushed with dry nitrogen and the reaction mixture was heated to 300 °C. Once the temperature was reached, 0.2 mL of 1 M TOP:Se solution was swiftly injected into the reaction mixture initiating nanosheet growth and left for 3 h. Finally, the reaction was quenched by rapid cooling to room temperature, and WSe_2_ nanosheets were precipitated by centrifugation at 5000 rpm for 10 min and washed several times with acetone. Black precipitate was finally redispersed in 5 mL of ethanol. In order to grow 1T’ WSe_2_ nanosheets on arbitrary conductive substrate, a piece of either CP, CF or Au foil (4 cm^2^) was placed in the flask prior to selenium precursor injection.

### 1T’/2H phase conversion

As-produced 1T’ WSe_2_ nanoflowers were drop-cast on Si/SiO_2_ substrates and then annealed in a tube furnace at 400 °C for 2 h in argon atmosphere (0.45 mbar). Annealed WSe_2_ nanoflowers were then redispersed in ethanol.

### Transmission electron microscopy

Samples for TEM analysis were prepared on carbon-coated copper grids. Preliminary low-magnification TEM imaging was performed using a JEOL JEM-2100Plus microscope. HR TEM images and ADF STEM images were acquired on a JEOL JEM-2100F microscope with a field-emission gun operated at 200 kV accelerating voltage.

### Scanning electron microscopy

SEM imaging and elemental analysis were performed using a Zeiss LEO Gemini 1525 field emission scanning electron microscope equipped with an Oxford x-act PentaFET Precision EDS detector. Images were acquired at 5 kV accelerating voltage at working distances <10 mm, while EDS maps were obtained at 20 kV.

### X-ray photoelectron spectroscopy

Measurements were performed on a Thermo Fisher K-Alpha^+^ spectrometer equipped with a monochromated micro-focused Al K_α_ X-ray source. The measurements were performed at room temperature using a 20 eV energy pass with energy step of 0.1 eV. Binding energies were calibrated using a C 1*s* peak at 284.6 eV. XPS spectra were analysed using the Avantage software package.

### Powder XRD

Concentrated suspensions of 1T’ WSe_2_ nanosheets were drop-cast on Si/SiO_2_ substrates. X-ray diffractograms were acquired on a Bruker D2 Phaser diffractometer equipped with the Cu source and operating in the reflection scan geometry. Diffraction patterns were collected in the range 6–50 degrees 2*θ* with a step size of 0.026 degrees. Small-angle XRD was performed on a PANlytical X’PERT-PRO X-ray diffractometer (Cu K_α_ source) in the range 3–20 degrees 2*θ* with a step size of 0.03 degrees. Crystal structures and reference diffraction patterns of the 1T’ phase of WSe_2_ were simulated in the CrystalMaker and CrystalDiffract software packages based on either orthorhombic WTe_2_ (space group *Pnm2*_*1*_) or monoclinic MoTe_2_ (space group *P2*_*1*_*/m*), cell parameters *a* = 5.76 Å and *b* = 3.30 Å were estimated from the results of HR TEM and *c* parameter was adjusted to fit (002) peak in the experimental diffractogram and found to be approximately 12.9 Å.

### Raman spectroscopy

Raman spectra were recorded using a Renishaw inVia Qontor confocal Raman microscope at excitation wavelength of 532 nm using an 1800 lines mm^−1^ grating. Laser power was kept under 0.195 mW (1%) to avoid damage to the sample.

### UV-vis spectroscopy

Optical spectroscopy was performed using a PerkinElmer Lambda 25 UV-vis spectrometer in 1 cm path quartz cuvettes. Absorption spectra were recorded between 1100 and 200 nm with 1 nm spectral resolution.

### Kelvin probe force microscopy

Atomic force microscopy and KPFM were performed using an Asylum MFP-3d microscope equipped with a PtIr-coated Silicon probe (Bruker SCM-PIT-V2). Typical initial scan parameters were 16 μm^2^ scan size, 256 points per line and a scan rate of 1 Hz. Higher-resolution scans were performed with 1024 points per line and scan rate of 1 Hz. Contact potential difference between the sample and the KPFM tip can be calculated using the equation ${\mathrm{CPD}} = \frac{{\phi _{{\mathrm{tip}}} - \phi _{{\mathrm{sample}}}}}{e}$, where *e* is the electron charge. Samples for KPFM were prepared on p-doped Si wafers; p-Si was used as a reference. Work function of WSe_2_ *ϕ*_Wse2_ was calculated with respect to the work function of p-doped Si *ϕ*_Si_ following the equation $\phi _{{\mathrm{WS}}_2} = \phi _{{\mathrm{Si}}} - e \times ({\mathrm{CPD}}_{{\mathrm{WS}}_2} - {\mathrm{CPD}}_{{\mathrm{Si}}})$.

### Differential scanning calorimetry

DSC was conducted using a Mettler Toledo DSC 3 calorimeter in nitrogen atmosphere in the temperature range 50–450 °C at heating rate of 20° min^−1^.

### Inductively coupled plasma emission spectroscopy

Qualitative elemental analysis was carried out on a Thermo Scientific iCAP 6000 Series spectrometer. Samples for ICP ES were digested in a 45% HNO_3_ solution and then diluted to analytical concentrations in Milli-Q water.

### Electrochemical characterisation (EC)

EC testing was performed in a standard three-electrode cell configuration with a carbon foil (99.8%, Aldrich) counter electrode and a commercial Ag/AgCl (3 M KCl) reference electrode (BASi, MF-2052). In all, 1 M H_2_SO_4_ (95%, VWR) solution in Milli-Q water was chosen as electrolyte. Geometric active area of working electrodes varied from 0.25 to 0.3 cm^2^. All potentials were applied vs Ag/AgCl (3 M KCl) reference electrode and were converted to vs Reversible Hydrogen Electrode (RHE) following the equation *V*(vs RHE) = *V*(vsAg/AgCl) + 0.059 × pH + 0.21. Polarisation curves were recorded in the range from 0 to −0.8 V vs reference electrode at a scan rate 5 mV s^−1^. EIS was performed at a bias potential of −0.5 V vs Ag/AgCl (3 M KCl) while sweeping the frequency from 1 MHz to 10 mHz with an amplitude of 20 mV. The EIS data were fitted to a Randles equivalent circuit.

## Supplementary information

Supplementary Information

## Data Availability

The data that support the findings of this study are available from the corresponding author upon request.
