# Supplementary material for: Direct solution-phase synthesis of 1T’ WSe2 nanosheets
Source: Nat Commun. 2019 Feb 12;10:712. doi: 10.1038/s41467-019-08594-3 (PMC6372596; doi:10.1038/s41467-019-08594-3)
Supplement: Supplementary file 1 — Supplementary Information [file 41467_2019_8594_MOESM1_ESM.pdf]

SUPPLEMENTARY INFORMATION

Direct Solution-Phase Synthesis of 1T' WSe_2 Nanosheets

Sokolikova et al.

Supplementary Figure 1. Structural polymorphs of WSe_2. 3
Supplementary Figure 2. Gallery of additional TEM images. 4
Supplementary Figure 3. Edge termination of individual 1T' WSe_2 nanosheets 5
Supplementary Figure 4. Effect of the growth conditions on the morphology and crystal structure of the colloidal WSe_2 nanosheets. 6
Supplementary Figure 5. Effect of additives on the morphology and crystal structure of the WSe_2 nanosheets. 7
Supplementary Figure 6. Absorption spectra of the aliquots taken at late stages of the reaction performed at various concentrations of the molecular precursor. 8
Supplementary Figure 7. Surface charge of the as produced 1T' and the annealed 2H WSe_2 nanoflowers. 9
Supplementary Figure 8. Stability of the 1T' phase under ambient conditions in solution. 10
Supplementary Figure 9. DSC study of the 1T' to 2H structural phase transition. 11
Supplementary Figure 10. Effect of the annealing at 400 °C on the morphology and crystal structure of the colloidal WSe_2 nanosheets. 12
Supplementary Figure 11. Effect of the pre-annealing at 200 °C on the crystal structure of the produced WSe_2 nanosheets. 13
Supplementary Figure 12. Small-angle diffraction study of the colloidal WSe_2 nanosheets. 14
Supplementary Figure 13. Formation of dense arrays of the 1T' WSe_2 nanosheets on various conductive substrates. 15
Supplementary Figure 14. Stability of the 1T' phase under electrocatalytic testing conditions. 16
Supplementary Figure 15. Catalyst mass loading effect on the performance of the working electrode. 17
Supplementary Figure 16. Comparison plot summarising the reported group VI TMD electrocatalysts for hydrogen evolution reaction. 18
Supplementary References 19

Supplementary Figures

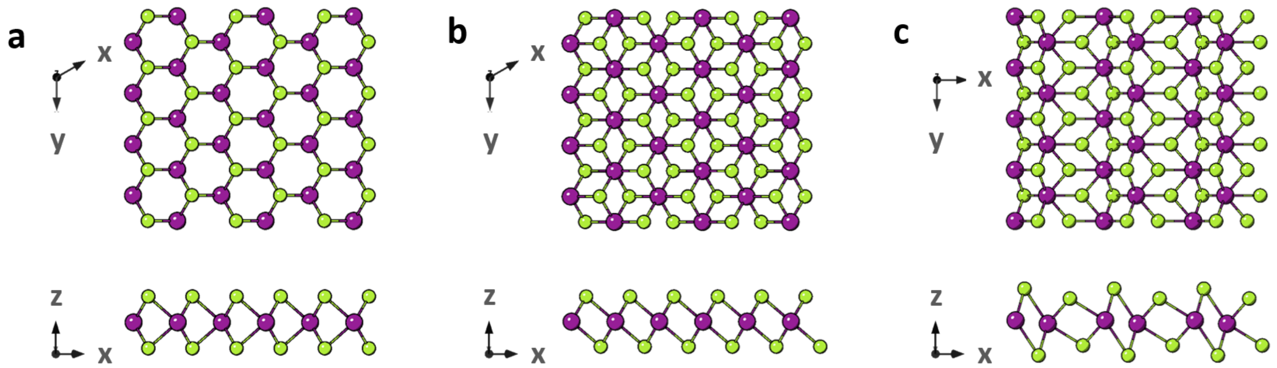

Supplementary Figure 1. Structural polymorphs of WSe_2. a – 2H WSe_2 (space group *$P6_3/mmc$*). **b** – 1T WSe_2 (space group *P-3m1*). **c** – 1T' WSe_2 (space group *$P2_1/m$*).

Atomic structures of the 1T and 1T' monolayers are simulated in the CrystalMaker software based on the structural parameters estimated from the HR TEM results. In the crystal models, purple circles represent tungsten atoms, green circles represent selenium atoms.

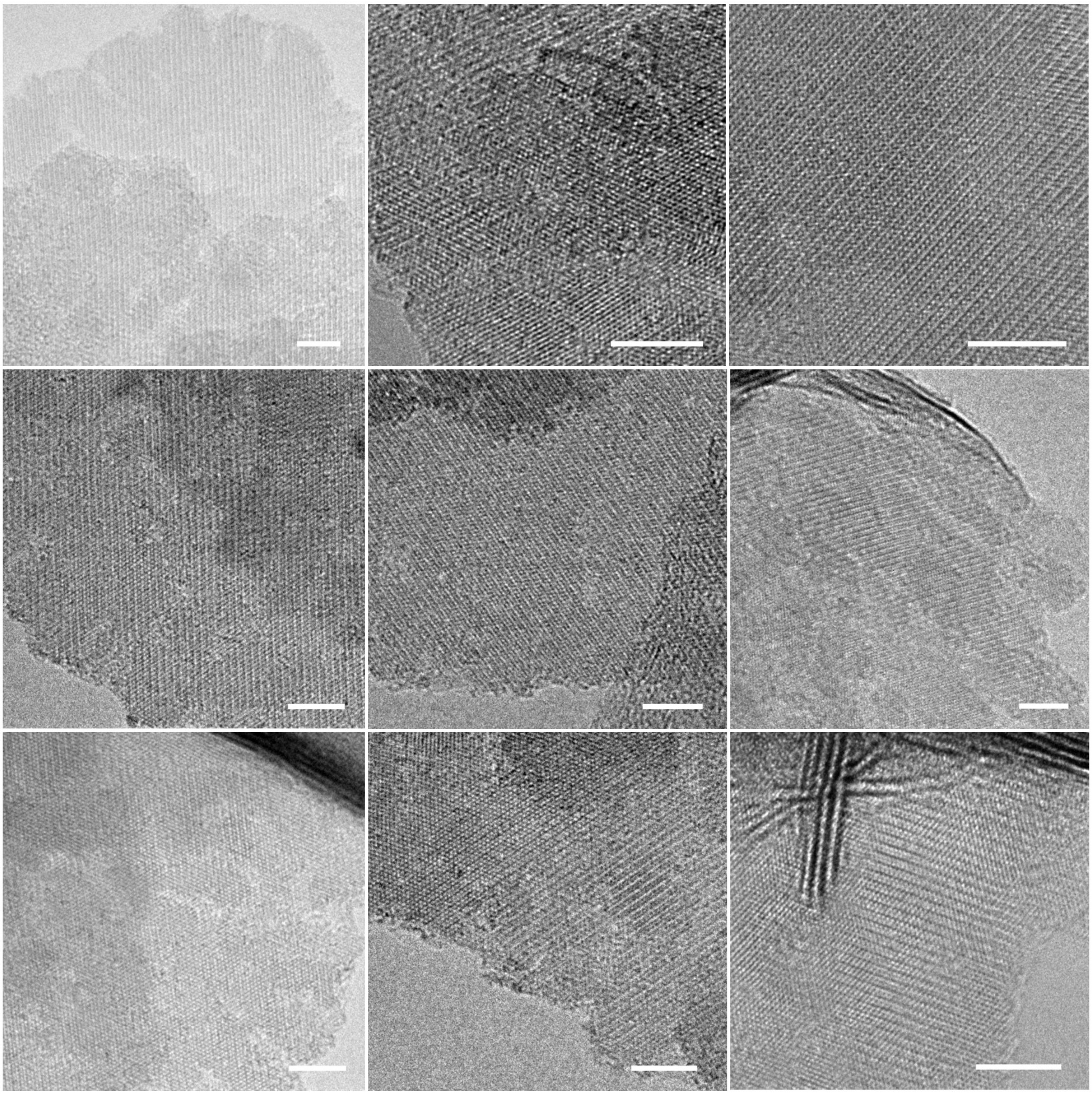

Supplementary Figure 2. Gallery of additional TEM images.

Additional HR TEM images are provided to demonstrate the continuous 1T’ phase reproducibly obtained over large areas in the synthesis of the WSe_2 nanoflowers. Scale bar 5 nm.

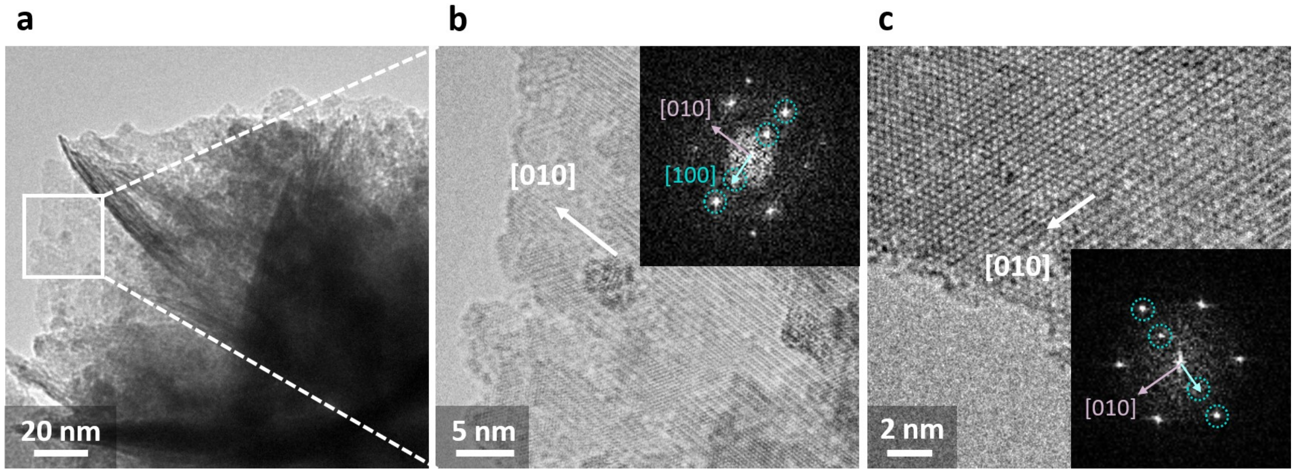

Supplementary Figure 3. Edge termination of individual 1T' WSe_2 nanosheets. a – low-magnification TEM image of a single petal of a 1T' WSe_2 nanoflower. **b, c** – high-resolution TEM images of petal edges indicating the [010] as a growth direction.

Rigid zigzag chains of tungsten atoms running through the flexible WSe_2 nanosheets cause scrolling of the individual petals predominantly along the [010] direction thus leading to a significant lattice compression in the [100] direction.

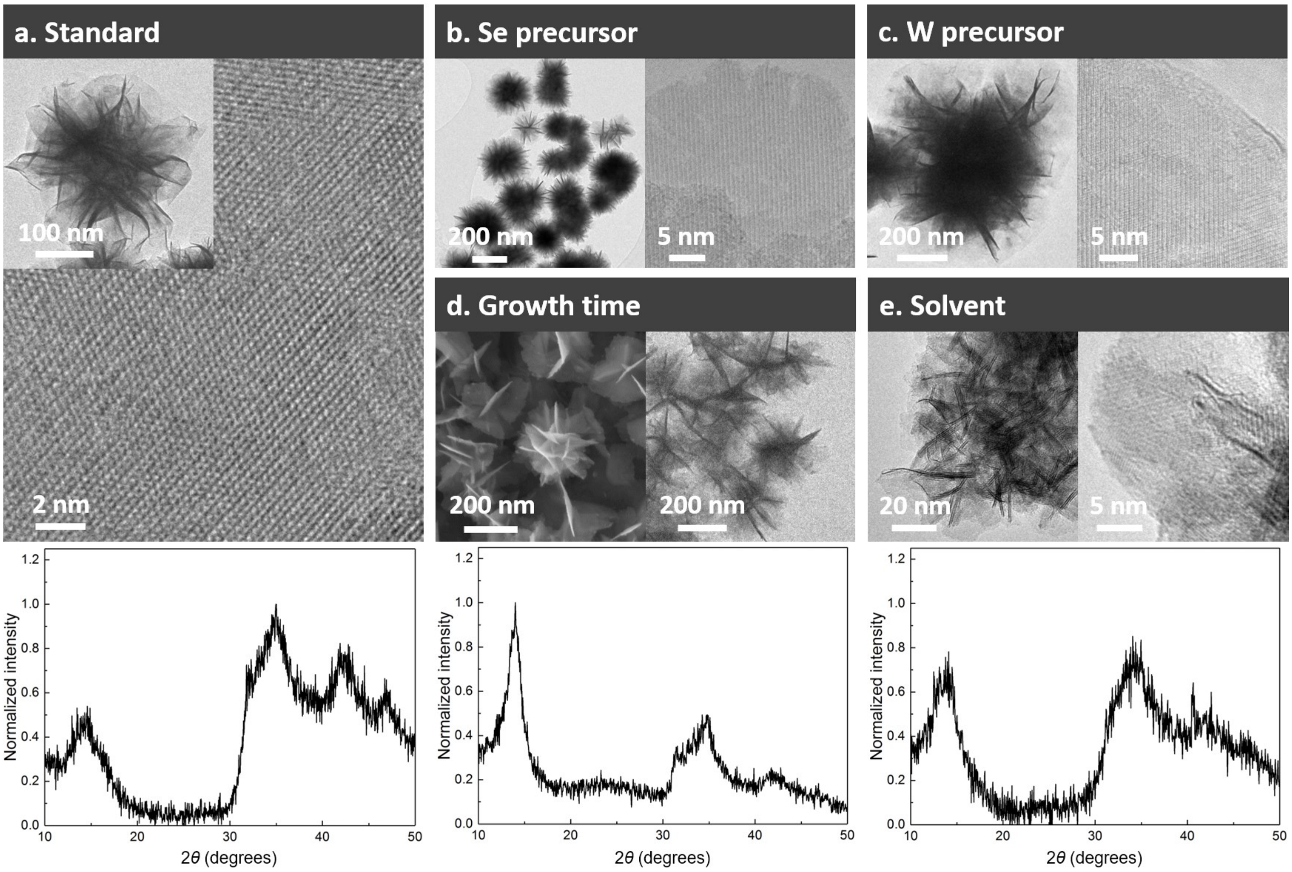

Supplementary Figure 4. Effect of the growth conditions on the morphology and crystal structure of the colloidal WSe_2 nanosheets. **a** – standard synthesis of the 1T' WSe_2 nanosheets. **b** – selenium precursor is changed to more active elemental selenium. **c** – tungsten precursor is changed to more active WCl_6. **d** – synthesis time extended to 15 hours. **e** – coordinating solvent is changed to weakly binding oleylamine.

In a standard synthesis, 1T' WSe_2 branched nanosheets are obtained through the reaction between $W(CO)_6$ and 1M TOP:Se at 300 °C in oleic acid (a). Change in chalcogen or metal precursor to more reactive elemental selenium (b) or WCl_6 (c), respectively, led to formation of denser nanoflowers, however no variation in crystal structure was observed. Conducting the growth in oleylamine instead of oleic acid resulted in formation of the branched nanosheets with smaller petals, however 1T' crystal structure was preserved (XRD). Increase in the synthesis time from 3 to 15 hours (d) led to formation of the WSe_2 nanoflowers with thicker petals as evidenced by the increased intensity of the (002) reflection in the XRD pattern. Absence of a strong (100) peak at 31 degrees 2θ, which is characteristic to the 2H phase, suggests the retention of the 1T' phase even after 15 hours at 300 °C in solution.

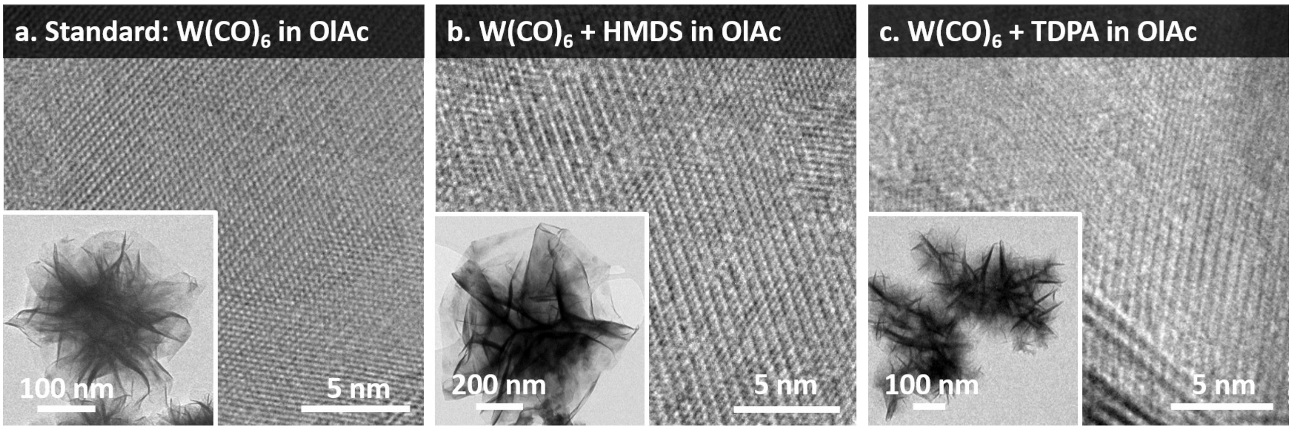

Supplementary Figure 5. Effect of additives on the morphology and crystal structure of the WSe_2 nanosheets. a – standard synthesis of the 1T' WSe_2 nanosheets. **b** – 1T' WSe_2 nanosheets obtained in presence of hexamethyldisilazane. **c** – 1T' WSe_2 nanosheets obtained in presence of tetradecylphosphonic acid.

Two additives, namely hexamethyldisilazane (HMDS, b) and tetradecylphosphonic acid (TDPA, c), were used to modify the reactivity of tungsten complex in oleic acid through partial complexing of tungsten atoms and substitution of oleate groups. HMDS is expected to increase the reactivity of tungsten complex due to weaker binding to a transition metal compared to carboxylic acid [1], while TDPA, being a stronger binding ligand than oleic acid [2], is presumed to lower the reactivity of tungsten precursor. According to [1] (reference 15 in the manuscript), higher reactivity of metal precursor causes formation of the thermodynamically stable 2H phase, however we found that in case of the WSe_2 nanosheets such alteration of the reactivity of tungsten complex did not affect the phase obtained. We should point out that the less reactive tungsten precursor (b) led to formation of larger WSe_2 nanoflowers of up to 800 nm in diameter compared to smaller, 200-300 nm in diameter, nanoflowers grown in a standard synthesis yet revealing the same ultra-thin nature of individual petals, while the presence of strongly binding TDPA (c) caused formation of dense nanoflowers (higher degree of branching).

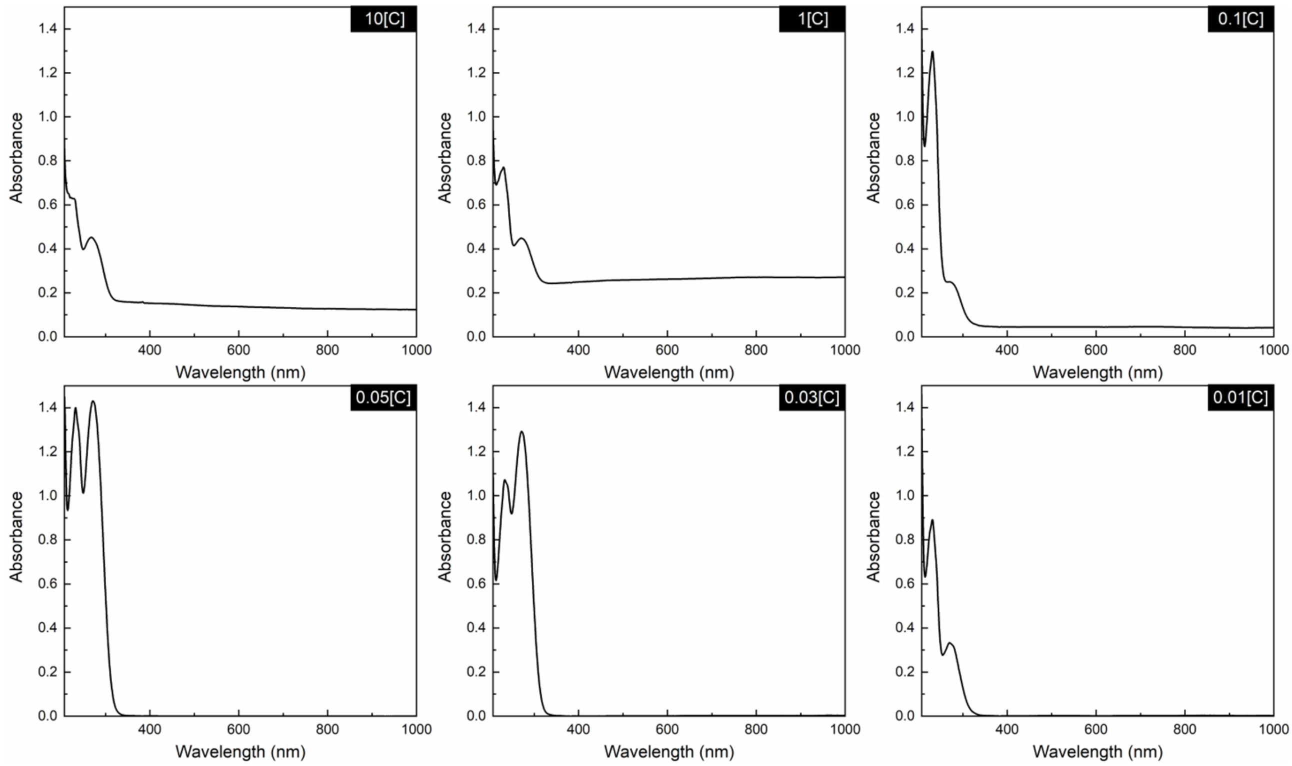

Supplementary Figure 6. Absorption spectra of the aliquots taken at late stages of the reaction performed at various concentrations of the molecular precursor.

Absorption spectroscopy study of the aliquots taken at different stages into the reaction show strong absorption bands (~260 nm and ~280 nm) of free oleic acid used as solvent obscuring any changes in concentration of the tungsten complex (absorption at ~280 nm) being consumed during the reaction due to the little amount of precursors (precursor concentration [C] in a standard synthesis is as low as 7.5 mmol L^{-1}). Additionally, being metallic in nature 1T' WSe_2 does not exhibit any excitonic features in the visible range, hindering the detection of the material at early stages of growth until the lateral size of the nanoflowers is big enough to cause a detectable scattering in the visible range.

We have varied the precursor concentration in the broad range 10[C]-0.01[C]. Presented here are the absorption spectra of the aliquots taken at late stages of the reaction. Observable scattering is detected only in the range of concentrations 10[C]-0.1[C] and correspond to 1T' phase of WSe_2, nucleation was not detected at lower precursor concentrations (0.05[C]-0.01[C]), presumably due to a small amount of the material.

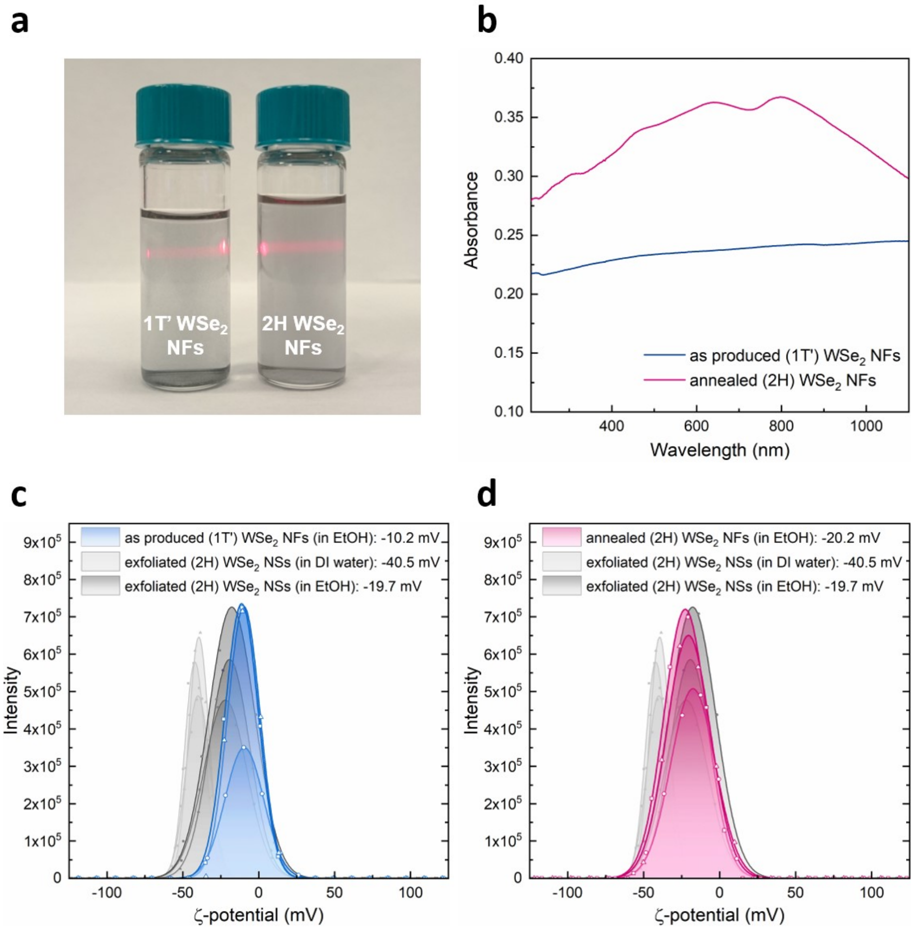

Supplementary Figure 7. Surface charge of the as produced 1T' and the annealed 2H WSe_2 nanoflowers. **a** – photograph of the stable 1T' and 2H WSe_2 nanoflowers (NFs) suspensions in ethanol. **b** - optical absorption spectra of the WSe_2 NFs before and after annealing proving the 1T' to 2H phase conversion. **c, d** - ζ-potentials of the suspensions of the as produced 1T' WSe_2 NFs (c, blue), annealed 2H WSe_2 NFs (d, purple), and 2H WSe_2 nanosheets (NSs) exfoliated in liquid phase (grey) as control.

Since as synthesised WSe_2 nanoflowers were found to be insoluble in water, stable suspensions in ethanol were studied instead. Negative potentials (average of three experiments) are measured for both synthesised 1T' WSe_2 nanoflowers and exfoliated 2H WSe_2 nanosheets used as control. Thus the inherent negative surface charge of both systems can be possibly attributed to the presence of chalcogen vacancies in the basal plane of the two-dimensional sheets [3]. Dispersions of 2H WSe_2 nanosheets produced via liquid-phase exfoliation in ethanol and in DI water exhibit negative ζ-potentials of -19.7 and -40.5 mV, respectively; suspension of the freshly prepared 1T' WSe_2 nanoflowers shows the ζ-potential of -10.2 mV (c), while a suspension of the annealed WSe_2 nanoflowers is characterised by slightly lower potential of -20.2 mV (d). The increase in the potential absolute value can be caused by a minor loss of selenium occurring during the annealing.

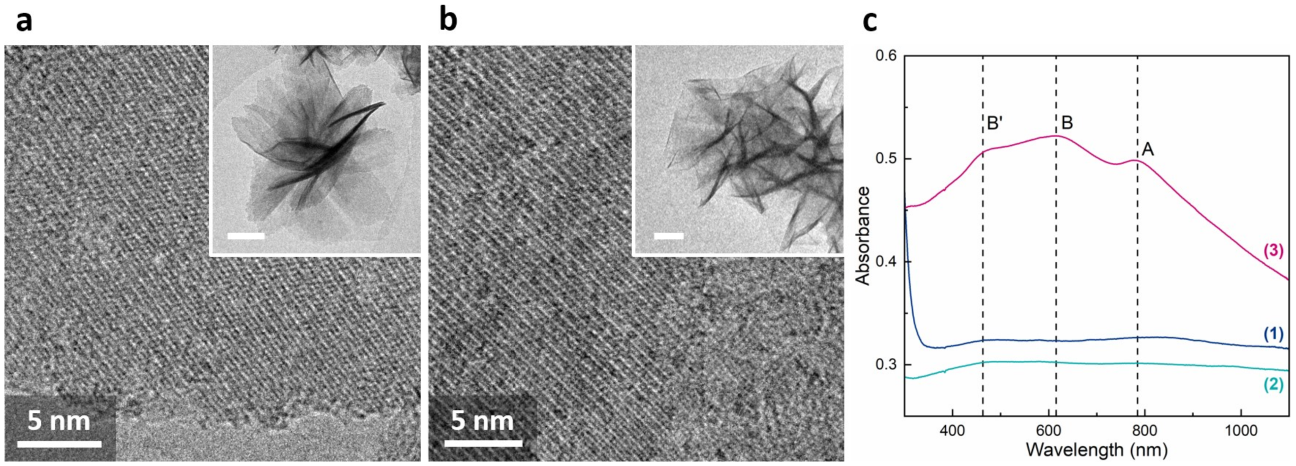

Supplementary Figure 8. Stability of the 1T' phase under ambient conditions in solution. a, b – high resolution TEM images of the WSe_2 nanosheets taken on the 1st day and the 15th day after the synthesis, respectively. Representative TEM images of nanoflowers are shown in insets (scale bar 50 nm). **c** – UV-vis spectra of the as produced (1) and stored in ethanol over a month (2) 1T' WSe_2 nanoflowers; absorption spectrum of the thermally converted 2H WSe_2 nanoflowers is presented for comparison (3).

Samples for UV-vis spectroscopy were prepared by diluting 100 µL aliquots in 1 mL of ethanol; lower signal recorded for the sample (2) can be caused by partial aggregation of the WSe_2 nanoflowers over a month in solution.

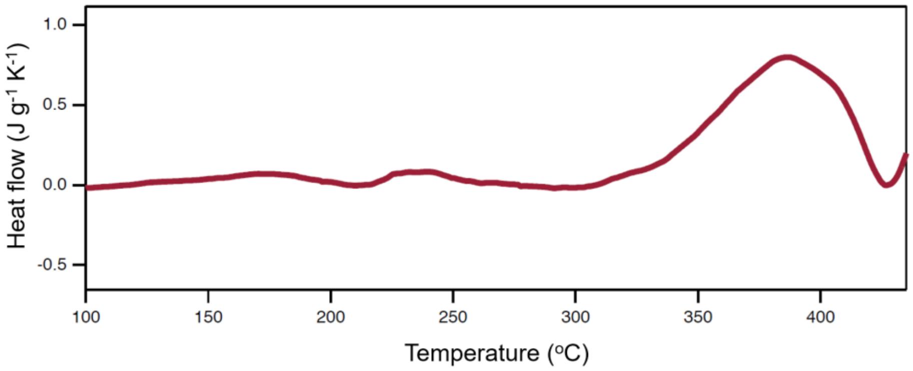

Supplementary Figure 9. DSC study of the 1T' to 2H structural phase transition.

DSC curve shows an exothermic peak centred at 395 ºC corresponding to the 1T' to 2H phase transition in the WSe_2 nanoflowers.

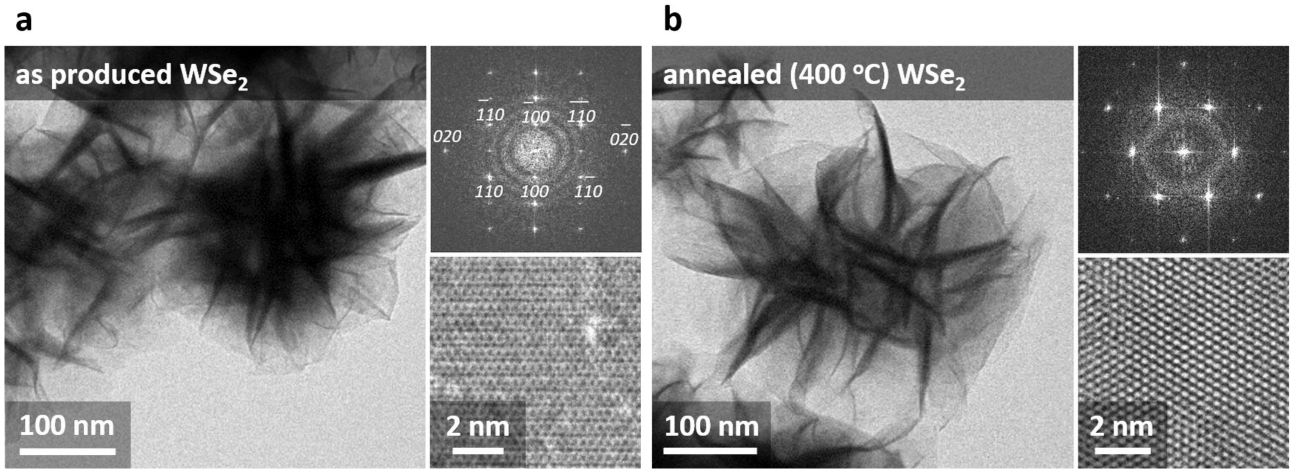

Supplementary Figure 10. Effect of the annealing at 400 ºC on the morphology and crystal structure of the colloidal WSe_2 nanosheets. a, b – TEM images of the individual nanoflowers, HRTEM images and the corresponding FFT patterns of the as produced 1T' WSe_2 and the 2H WSe_2 nanoflowers obtained via annealing at 400 ºC, respectively.

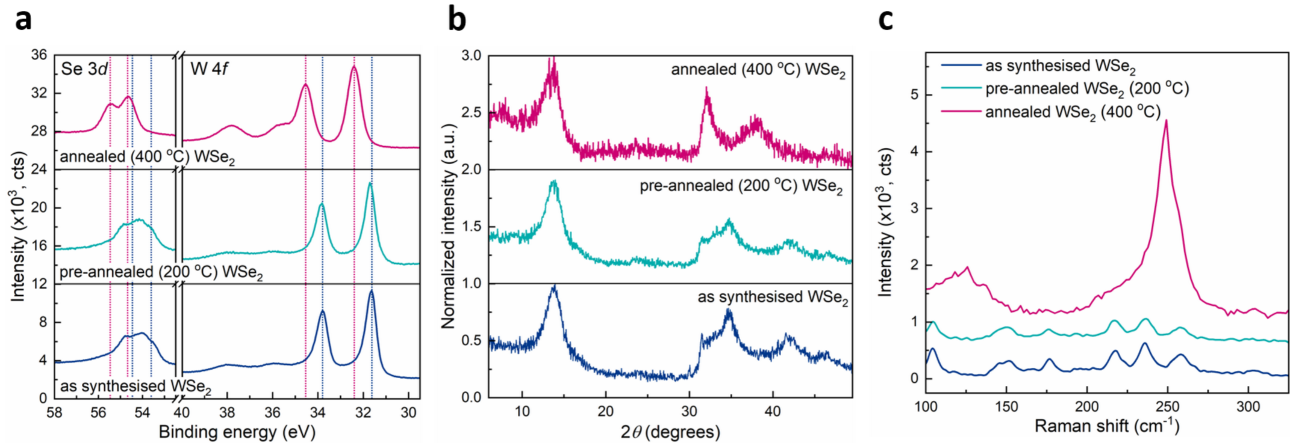

Supplementary Figure 11. Effect of the pre-annealing at 200 ºC on the crystal structure of the produced WSe_2 nanosheets. a – XPS spectra, **b** – X-ray diffractograms, and **c** – Raman spectra of the pristine 1T' (bottom row), annealed at 200 ºC (middle row) and fully converted into 2H via annealing at 400 ºC (top row) WSe_2 nanoflowers.

Results of the XPS, XRD, and Raman spectroscopy confirm that pre-annealing at 200 ºC does not induce 1T' to 2H phase transition in the WSe_2 nanoflowers.

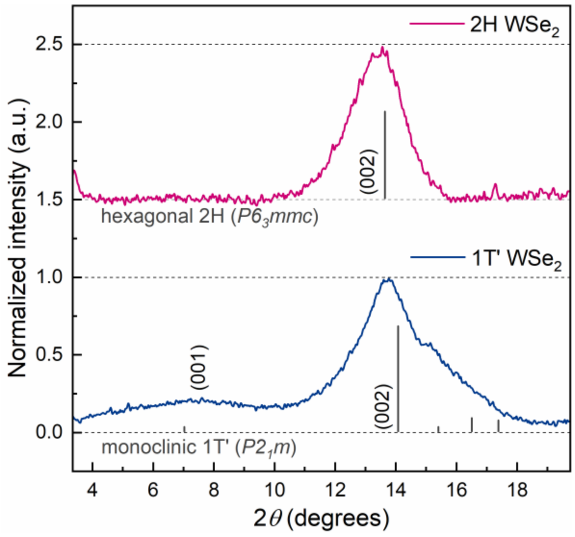

Supplementary Figure 12. Small-angle diffraction study of the colloidal WSe_2 nanosheets. Small-angle XRD patterns of the as produced 1T' WSe_2 (bottom) and annealed 2H WSe_2 (top) nanosheets.

Broad peak centred at 7 degrees 2θ is assigned to the (001) reflection of the monoclinic 1T' phase (space group $P2_1/m$).

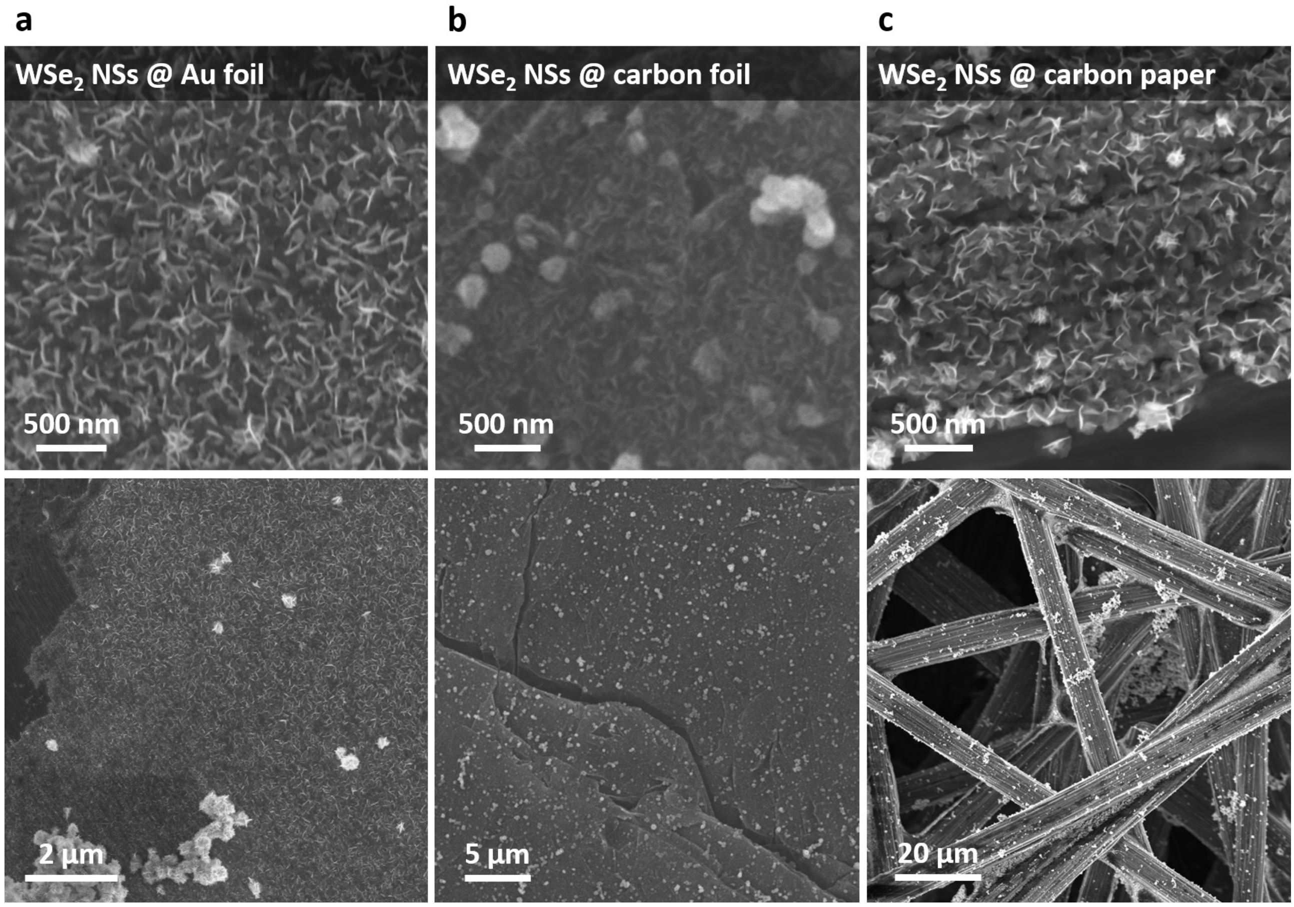

Supplementary Figure 13. Formation of dense arrays of the 1T' WSe_2 nanosheets on various conductive substrates. Representative SEM images of the vertically aligned 1T' WSe_2 nanosheets grown directly on **a** – gold foil, **b** – on carbon foil, and **c** – on carbon paper.

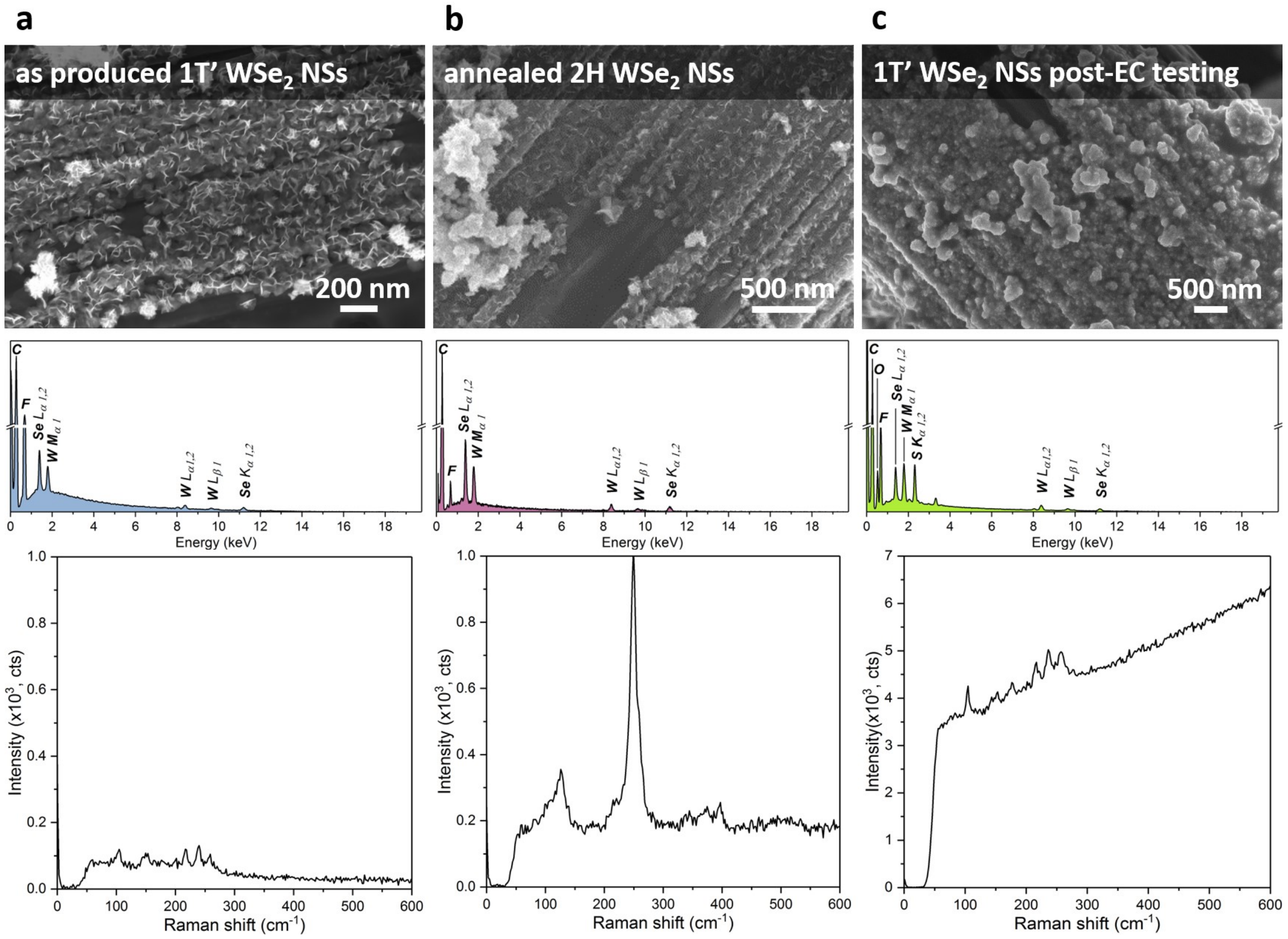

Supplementary Figure 14. Stability of the 1T’ phase under electrocatalytic testing conditions. SEM images, EDS and Raman spectra of the WSe_2 nanosheets grown on carbon paper: **a** – as produced 1T’; **b** – annealed (400 °C) 2H; and **c** – after LSV cycling (300 cycles) in the range from 0 to -0.8 V vs Ag/AgCl reference electrode.

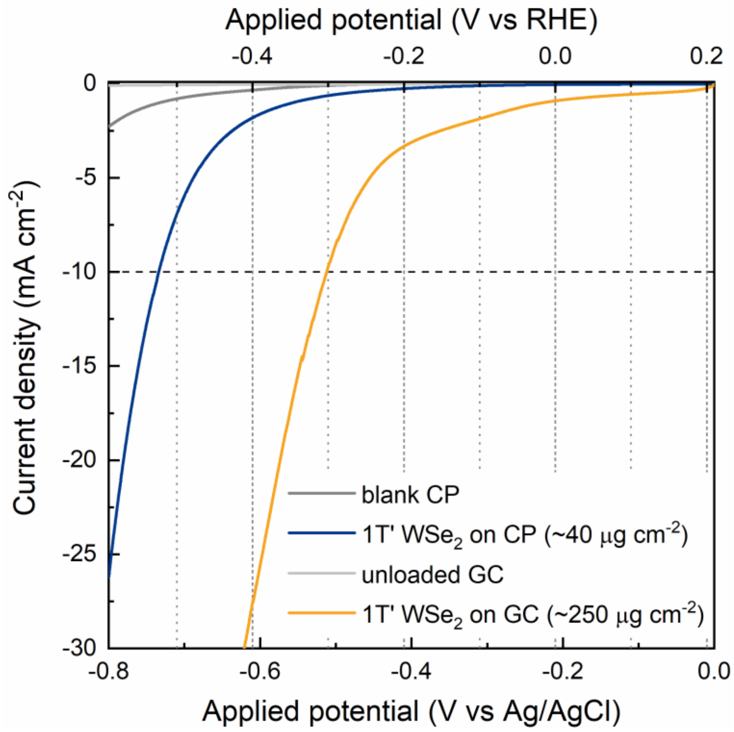

Supplementary Figure 15. Catalyst mass loading effect on the performance of the working electrode. Polarisation curves of the 1T' WSe_2 nanosheets working electrodes with different catalyst mass loading.

Compared here are polarisation curves of two working electrodes with different mass loadings of the 1T' WSe_2 nanosheets illustrating a clear decrease in overpotential from 510 mV to 300 mV when catalyst mass loading increases from 40 μg cm^{-2} (material grown directly on carbon paper) to 250 μg cm^{-2} (drop-cast on a glassy carbon electrode).

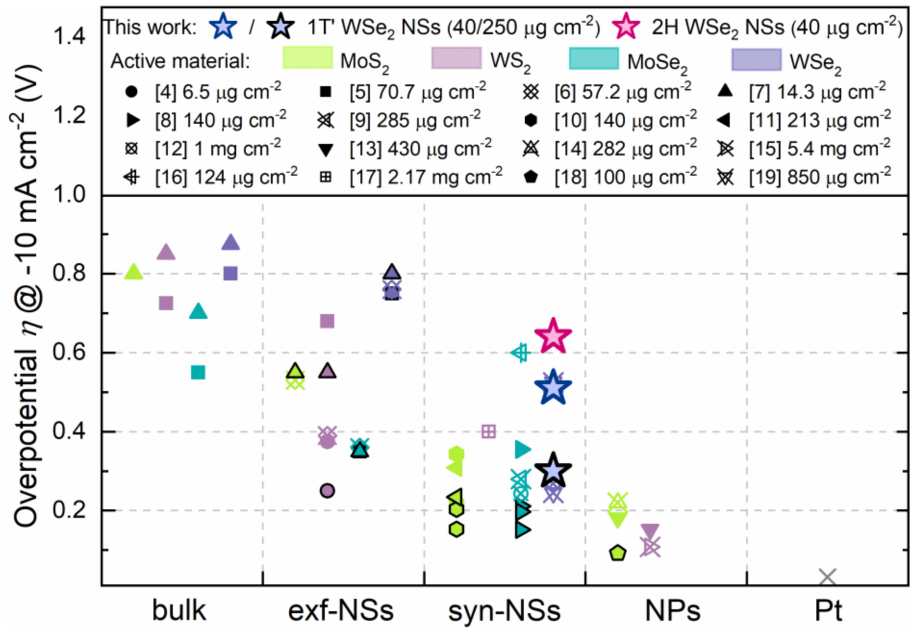

Supplementary Figure 16. Comparison plot summarising the reported group VI TMD electrocatalysts for hydrogen evolution reaction.

Catalyst mass loading is stated in the legend for each reference data point are taken from ref [4,5,14–19,6–13] (references 7 and 36-50 in the manuscript). Overpotentials, at which the working electrodes composed of the colloidal 1T’ or 2H WSe_2 nanosheets (mass loading 40 µg cm^{-2}) presented in the manuscript reach current density of - 10 mA cm^{-2}, are shown as filled stars (blue and purple, respectively). To illustrate the effect of the mass loading of active material on overall performance of the working electrode, we have also included a third experimental data point (blue star with black border) corresponding to the colloidal 1T’ WSe_2 nanosheets drop-cast onto a glassy carbon electrode to achieve a significantly higher mass loading (250 µg cm^{-2}).

Supplementary References

1. Mahler, B., Hoepfner, V., Liao, K. & Ozin, G. A. Colloidal synthesis of 1T-WS2 and 2H-WS2 nanosheets: Applications for photocatalytic hydrogen evolution. *J. Am. Chem. Soc.* **136**, 14121–14127 (2014).
2. Puzder, A. *et al.* The effect of organic ligand binding on the growth of CdSe nanoparticles probed by ab initio calculations. *Nano Lett.* **4**, 2361–2365 (2004).
3. Park, J. H. *et al.* Defect passivation of transition metal dichalcogenides via a charge transfer van der Waals interface. *Sci. Adv.* **3**, e1701661 (2017).
4. Voiry, D. *et al.* Enhanced catalytic activity in strained chemically exfoliated WS2 nanosheets for hydrogen evolution. *Nat. Mater.* **12**, 850–855 (2013).
5. Eng, A. Y. S., Ambrosi, A., Sofer, Z., Šimek, P. & Pumera, M. Electrochemistry of transition metal dichalcogenides: Strong dependence on the metal-to-chalcogen composition and exfoliation method. *ACS Nano* **8**, 12185–12198 (2014).
6. Chia, X., Ambrosi, A., Sofer, Z., Luxa, J. & Pumera, M. Catalytic and charge transfer properties of transition metal dichalcogenides arising from electrochemical pretreatment. *ACS Nano* **9**, 5164–5179 (2015).
7. Tan, S. M., Sofer, Z., Luxa, J. & Pumera, M. Aromatic-exfoliated transition metal dichalcogenides: Implications for inherent electrochemistry and hydrogen evolution. *ACS Catal.* **6**, 4594–4607 (2016).
8. Yin, Y. *et al.* Synergistic phase and disorder engineering in 1T-MoSe2 nanosheets for enhanced hydrogen-evolution reaction. *Adv. Mater.* **29**, 1700311 (2017).
9. Zhou, X. *et al.* Fast colloidal synthesis of scalable Mo-rich hierarchical ultrathin MoSe(2−x) nanosheets for high-performance hydrogen evolution. *Nanoscale* **6**, 11046–11051 (2014).
10. Yin, Y. *et al.* Contributions of phase, sulfur vacancies, and edges to the hydrogen evolution reaction catalytic activity of porous molybdenum disulfide nanosheets. *J. Am. Chem. Soc.* **138**, 7965–7972 (2016).
11. Wang, D. *et al.* Phase engineering of a multiphasic 1T/2H MoS 2 catalyst for highly efficient hydrogen evolution. *J. Mater. Chem. A* **5**, 2681–2688 (2017).
12. Meiron, O. E., Kuraganti, V., Hod, I., Bar-Ziv, R. & Bar-Sadan, M. Improved catalytic activity of Mo(1−x)W(x)Se2 alloy nanoflowers promotes efficient hydrogen evolution reaction in both acidic and alkaline aqueous solutions. *Nanoscale* **9**, 13998–14005 (2017).

13. Zhou, M. *et al.* Colloidal preparation and electrocatalytic hydrogen production of MoS2 and WS2 nanosheets with controllable lateral sizes and layer numbers. *Nanoscale* **8**, 15262–15272 (2016).

14. Vrubel, H., Moehl, T., Grätzel, M. & Hu, X. Revealing and accelerating slow electron transport in amorphous molybdenum sulphide particles for hydrogen evolution reaction. *Chem. Commun.* **49**, 8985 (2013).

15. Zhou, H. *et al.* Highly efficient hydrogen evolution from edge-oriented WS2(1– x)Se2x particles on three-dimensional porous NiSe2 foam. *Nano Lett.* **16**, 7604–7609 (2016).

16. Zhang, C. *et al.* Phosphine-free synthesis and shape evolution of MoSe 2 nanoflowers for electrocatalytic hydrogen evolution reactions. *CrystEngComm* **20**, 2491–2498 (2018).

17. Huang, G. *et al.* Hierarchical architecture of WS 2 nanosheets on graphene frameworks with enhanced electrochemical properties for lithium storage and hydrogen evolution. *J. Mater. Chem. A* **3**, 24128–24138 (2015).

18. Chen, W. *et al.* Quantum Dots of 1T Phase Transitional Metal Dichalcogenides Generated via Electrochemical Li Intercalation. *ACS Nano* **12**, 308–316 (2018).

19. Sun, Y., Zhang, X., Mao, B. & Cao, M. Controllable selenium vacancy engineering in basal planes of mechanically exfoliated WSe 2 monolayer nanosheets for efficient electrocatalytic hydrogen evolution. *Chem. Commun.* **52**, 14266–14269 (2016).
